# Age moderates the relationships between obesity, glucose variability, and intensive care unit mortality: a retrospective cohort study

**DOI:** 10.1186/s40560-021-00582-4

**Published:** 2021-10-26

**Authors:** Lusi Lu, Yifeng Lu, Chenlu Gao, Nan Zhang

**Affiliations:** 1 Department of Endocrinology, Sir Run Run Shaw Hospital, School of Medicine, Zhejiang University, 3 Qingchun E Rd, Hangzhou, Zhejiang China; 2 Touro College of Osteopathic Medicine, 60 Prospect Ave, Middletown, NY 10940 USA; 3 Division of Sleep and Circadian Disorders, Brigham and Women’s Hospital, 75 Francis St, Boston, MA 02115 USA; 4 Division of Sleep Medicine, Harvard Medical School, 221 Longwood Ave, Boston, MA 02115 USA

**Keywords:** Obesity paradox, Glycemic variability, Asian, Critical care, Elderly, Aging

## Abstract

**Background:**

Although the associations between obesity, glucose variability (GV), and Intensive Care Unit (ICU) mortality have been studied extensively, whether age moderates these associations is not well understood.

**Materials and methods:**

The medical records of 1062 patients, who were admitted into ICU at Sir Run Run Shaw Hospital (Zhejiang, China), were studied. Logistic regression was used to test the associations between obesity, GV, and ICU mortality. Furthermore, the moderation effect of age was tested.

**Results:**

After controlling for covariates, the underweight group had the highest odds of death (OR 2.38, 95% CI 1.43–3.95, *p* < 0.001) in comparison with the control group (overweight). However, normal weight (OR 1.29, 95% CI 0.88–1.89, *p* = 0.185) and obese (OR 1.08, 95% CI 0.61–1.90, *p* = 0.790) groups had similar odds of death, compared to the overweight group. Age significantly moderated the association between obesity and mortality, where being overweight was more advantageous than being normal weight in older adults (*B* = 0.03, SE = 0.01, OR 1.03, 95% CI 1.001–1.06, *p* = 0.045). Meanwhile, higher GV predicted greater mortality in adjusted models (OR 1.23, 95% CI 1.06–1.42, *p* = 0.005). We also found an interaction between age and GV (*B* = − 0.01, SE = 0.01, OR 0.99, 95% CI 0.98–0.999, *p* = 0.025), which suggested that the association between GV and mortality becomes weaker with increasing age.

**Conclusions:**

With increasing age, the association between BMI and mortality becomes stronger and the association between glucose variability and mortality becomes weaker. Future studies should investigate the underlying mechanisms of such phenomenon and the causal relationship between obesity, GV, and ICU mortality.

**Supplementary Information:**

The online version contains supplementary material available at 10.1186/s40560-021-00582-4.

## Background

Obesity is common and has been identified as a predictive factor for general health outcomes and hospital mortality among Intensive Care Unit (ICU) patients [1, 2]. In addition, glucose variability (GV) is a risk factor for the mortality among ICU patients that has been studied extensively in recent decades [3–5]. However, both obesity and GV are often ignored when estimating mortality in ICU patients (e.g., via calculating the Acute Physiology and Chronic Health Evaluation II [APACHE II] score). Furthermore, there are no clear guidelines on modifying treatment plans according to patients’ obesity status or GV levels.

Although the adverse effects of obesity on health outcomes have been studied extensively [1, 6, 7], a controversial phenomenon, namely, the obesity paradox, has not been well understood. The obesity paradox was seen in patients with chronic diseases (e.g., end-stage kidney disease, chronic obstructive pulmonary disease, heart failure) or critical acute illness (e.g., sepsis, acute cardiovascular disease), where hospital mortality rates were lower in overweight (i.e., body mass index [BMI] between 25 and 30 kg/m^2^) and obese (i.e., BMI between 30 and 35 kg/m^2^) patients compared to their normal-weight counterparts [8]. A meta-analysis review on the obesity paradox confirmed a U-shape association between obesity and mortality in patients undergoing cardiac surgeries, where the mortality rates were highest among underweight and extremely obese patients (BMI < 18 or > 35 kg/m^2^) and lowest among overweight patients [9]. Moreover, this phenomenon was also observed among critically ill patients [10].

However, the obesity paradox may not be present in all demographic groups [10, 11]. For example, Fukuoka et al. (2019) only observed the obesity paradox among elderly acute myocardial infarction patients in Japan, but not among younger patients with the same conditions, indicating that age might moderate the association between obesity and mortality [11]. Moreover, a recent study with more than 140,000 ICU patients (predominantly Caucasian) also reported that as age increases, the association between obesity and ICU mortality is strengthened [10]. Due to the globally rising trend of the aging population and obesity, it is important to investigate whether the association between obesity and mortality, as well as the moderation effect of age, can be found in other racial/ethnic groups.

In addition to obesity, lower blood glucose variability (GV) has been found to predict lower hospital mortality in critically ill patients [5]. In 2008, Krinsley reported that high standard deviations of blood glucose predicted high ICU mortality [5]. Later, Hermanides et al. (2010) found that low GV predicted low ICU mortality even in the patients whose mean glucose levels were high [4]. Although this association has been consistently observed, researchers have not investigated whether this association is similar across demographic groups, such as different age groups.

While many researchers agreed that aging is associated with insulin resistance [12], Xiao et al. (2013) found that non-diabetic older Chinese had greater insulin sensitivity than their younger counterparts even though beta-cell function was worse in the older patients [13]. Similar findings were reported in European and American populations [14, 15]. Despite the controversy on the relationship between aging and insulin sensitivity, these studies consistently demonstrated a decline in the ability to maintain glucose homeostasis during aging. It is unclear whether this change will affect the prognosis of critically ill elderly or affect the mechanisms underlying glucose variability and mortality. Therefore, it is important to investigate whether age moderates the association between GV and ICU mortality, to provide insights to tailored ICU glucose management.

In sum, we aimed to assess the associations between obesity, GV, and hospital mortality in ICU patients in China and to investigate whether such associations are moderated by age.

## Methods

### Participants

This is a single-center, retrospective, cohort study, which includes patients admitted to the ICU at Sir Run Run Shaw Hospital (Zhejiang, China) from July 1st, 2012 to September 30th, 2015. Patients with recent blood transfusions, hemoglobinopathies, and hemolysis were excluded due to the confounding effect on hemoglobin A1c levels. We used hemoglobin A1c level greater than 6.5% to confirm patients’ diabetes mellitus according to the American Diabetes Association 2021 guideline [16]. If a patient was re-admitted to the ICU within the same period, we only included the data from their first ICU stay. We additionally excluded patients with missing predictor or outcome data. The included 1062 patients were on average 63.08 years (age range 17–96; SD = 14.28) and 39.92% (*n* = 424) female. Power analyses suggest that this sample size yields 0.90 statistical power to detect small effect sizes (e.g., OR 1.30). Majority of the patients were admitted to the ICU due to gastrointestinal (*n* = 344; 32.39%), cardiovascular (*n* = 246; 23.16%), neurological (*n* = 195; 18.36%), or respiratory (*n* = 111; 10.45%) diseases. This study was approved by the Research Ethics Committee at Sir Run Run Shaw Hospital. Written informed consent was waived for this study due to the retrospective nature. All patient information was de-identified by a designated staff to ensure confidentiality.

### Data collection

Lipid panel levels [triglyceride, cholesterol, low-density lipoproteins (LDL), high-density lipoproteins (HDL)], weight, and height were measured and recorded within 24 h of ICU admission. Age, sex, diagnoses of diabetes mellitus, hypertension, duration of ICU stay, use of corticosteroids and vasopressors in the ICU, use of mechanical ventilation support, use of continuous renal replacement therapy (CRRT), and use of dialysis were extracted from medical history. Diagnoses of diabetes mellitus were also confirmed by patients’ hemoglobin A1c levels. Weight and height were used for the calculation of BMI:

$${\text{BMI}} = \frac{{{\text{Weight}}\left( {{\text{kg}}} \right)}}{{{\text{Height}}\left( {\text{m}} \right)^{2} }}$$

Because our study population is mainly Chinese residents, we adopted the criteria published by the National Health and Family Planning Commission of the People’s Republic of China to categorize BMI: underweight (BMI < 18.5 kg/m^2^), normal weight (BMI 18.5–23.99 kg/m^2^), overweight (BMI 24–27.99 kg/m^2^), and obese (BMI ≥ 28 kg/m^2^) [17].

Acute Physiology and Chronic Health Evaluation II (APACHE II) score was calculated for each patient within 24 h of ICU admission [18].

GV was quantified by the standard deviation of all glucose values during ICU stay [19]. All blood glucose measurements between ICU admission and discharge/death were used to compute mean glucose and GV levels. Blood glucose was measured once every 1 h to every 4 h depending on the patients’ condition. An ICU sliding scale insulin protocol was used for every ICU patient, regardless of diabetes status (for details see Additional file 1: Table S1). Hypoglycemia was defined as having a blood glucose measure at or below 3.9 mmol/L during ICU stay.

### Statistical analyses

We first conducted independent sample *t* tests (for continuous variables) and Chi-square tests (for categorical variables) to compare characteristics of survivors and non-survivors. The relationship between BMI/GV and covariates were examined through Pearson’s correlation or independent sample *t* tests.

Then, we conducted binary logistic regression to test whether BMI and GV predicted mortality in unadjusted and adjusted models. In the unadjusted models, BMI (either continuous or categorical) or GV (continuous) was included as the sole predictor and mortality was the outcome. In the adjusted models, a list of covariates were included in the model (age, sex, cholesterol, APACHE II score, diabetes mellitus, mean glucose levels, and use of corticosteroids) in addition to BMI (either continuous or categorical) or GV (continuous) as the predictors, while mortality was the outcome.

If BMI or GV was associated with mortality, we further tested whether such an association was moderated by age, after adjusting for the covariates. Using binary logistic regression models, we entered the covariates, BMI (either continuous or categorical) or GV (continuous), and the corresponding interaction term (BMI/GV with age) as predictors. The outcome in the models was mortality. Using the same approach, we additionally tested whether gender or cause of ICU admission moderated the association between BMI/GV and mortality.

All statistical analyses were performed using SPSS version 28. All tests were two-tailed with alpha set to 0.05. Note that age and GV were used as continuous variables in the statistical models; however, for visual illustration in figures, we categorized participants into three age groups (< 60, 60–69, ≥ 70) and five GV groups (i.e., quintiles).

## Results

### Patient characteristics

Characteristics of non-survivors and survivors are summarized in Table 1. Mortality rate was 29.28% among all patients. Compared to non-survivors, survivors were significantly younger and had higher BMI, lower 24-h APACHE II scores, higher total cholesterol, higher LDL, and lower GV (*p*s < 0.05). Moreover, use of mechanical ventilation, CRRT, corticosteroids, and vasopressors were more prevalent among non-survivors (*p*s < 0.05).

**Table 1:** Characteristics of non-survivors and survivors

	All (*N* = 1062)	Survivors (*n* = 751)	Non-survivors (*n* = 311)	*p* value	Effect size
Age	63.08 (14.28)	61.62 (13.74)	66.61 (14.96)	< 0.001***	*d* = 0.35
Sex (female)	424 (39.92%)	309 (41.15%)	115 (36.98%)	0.207	*Φ* = 0.04
BMI (kg/m^2^)	22.92 (4.23)	23.18 (4.11)	22.29 (4.44)	0.002**	*d* = 0.21
Underweight (BMI < 18.5)	146 (13.75%)	83 (11.05%)	63 (20.26%)	< 0.001***	*Φ* = 0.14
Normal weight (BMI 18.5–23.99)	520 (48.96%)	364 (48.47%)	156 (50.16%)		
Overweight (BMI 24–27.99)	282 (26.55%)	220 (29.29%)	62 (19.94%)		
Obese (BMI ≥ 28)	114 (10.73%)	84 (11.19%)	30 (9.65%)		
Reason for ICU admission					
Gastrointestinal causes	344 (32.39%)	270 (35.95%)	74 (23.79%)	< 0.001***	*Φ* = 0.23
Cardiovascular causes	246 (23.16%)	199 (26.50%)	47 (15.11%)		
Neurological causes	195 (18.36%)	128 (17.04%)	67 (21.54%)		
Respiratory causes	111 (10.45%)	56 (7.46%)	55 (17.68%)		
Other causes	166 (15.63%)	98 (13.05%)	68 (21.86%)		
APACHE II (first 24 h)	17.46 (8.24)	15.51 (7.08)	22.17 (8.93)	< 0.001***	*d* = 0.87
ICU stay length (days)	13.18 (28.54)	8.82 (14.88)	23.72 (45.76)	< 0.001***	*d* = 0.54
Triglyceride (mmol/L)	1.58 (2.98)	1.63 (3.41)	1.45 (1.49)	0.377	*d* = 0.06
Total cholesterol (mmol/L)	3.78 (1.53)	3.85 (1.48)	3.61 (1.62)	0.020*	*d* = 0.16
HDL (mmol/L)	0.87 (0.41)	0.88 (0.39)	0.83 (0.45)	0.074	*d* = 0.12
LDL (mmol/L)	2.10 (1.04)	2.15 (0.99)	1.97 (1.15)	0.009**	*d* = 0.18
Mean glucose (mmol/L)	10.16 (2.00)	10.19 (1.98)	10.09 (2.05)	0.471	*d* = 0.05
Blood glucose variability (standard deviation; mmol/L)	2.60 (1.18)	2.54 (1.17)	2.74 (1.19)	0.010*	*d* = 0.17
Hypoglycemia	142 (13.37%)	68 (9.05%)	74 (23.79%)	< 0.001***	*Φ* = 0.20
Diabetes mellitus	396 (37.29%)	293 (39.01%)	103 (33.12%)	0.071	*Φ* = 0.06
Hypertension	513 (48.31%)	355 (47.27%)	158 (50.80%)	0.303	*Φ* = 0.03
Hypertriglyceridemia	256 (24.11%)	190 (25.30%)	66 (21.22%)	0.256	*Φ* = 0.04
Hypercholesterolemia	148 (13.94%)	107 (14.25%)	41 (13.18%)	0.786	*Φ* = 0.01
Mechanical ventilation	772 (72.69%)	500 (66.58%)	272 (87.46%)	< 0.001***	*Φ* = 0.21
Dialysis	12 (1.13%)	10 (1.33%)	2 (0.64%)	0.333	*Φ* = 0.03
CRRT	193 (18.17%)	70 (9.32%)	123 (39.35%)	< 0.001***	*Φ* = 0.36
Corticosteroids	331 (31.17%)	171 (22.77%)	160 (51.45%)	< 0.001***	*Φ* = 0.28
Vasopressors	451 (42.47%)	234 (31.16%)	217 (69.77%)	< 0.001***	*Φ* = 0.36

Data presented as mean (standard deviation) or *n* (%). Effect sizes are Cohen’s d (for independent-sample *t* tests) or *Φ* (for Chi-square tests)

*APACHE* *II* Acute Physiology and Chronic Health Evaluation II; *BMI* body mass index; *CRRT* continuous renal replacement therapy; *HDL* high density lipoprotein; *ICU* intensive care unit; *LDL* low density lipoprotein

*p < 0.05, ** *p* < 0.01, *** *p* < 0.001

### Lower BMI is associated with higher mortality

First, we examined whether BMI was related to the selected covariates. Results showed that higher BMI was associated with younger age [*r*(1060) = − 0.13, *p* < 0.001], male sex [*t*(1060) = 2.09, *p* = 0.037], having diabetes mellitus [*t*(1060) = 4.77, *p* < 0.001], lower APACHE II score [*r*(1059) = − 0.09, *p* = 0.006], higher total cholesterol [*r*(1060) = 0.164, *p* < 0.001], and higher mean blood glucose [*r*(1060) = 0.185, *p* < 0.001]. BMI was not associated with use of corticosteroids [*t*(1060) = 0.32, *p* = 0.747].

We conducted binary logistic regression with BMI included in the model as a continuous variable. Patients with higher BMI had lower mortality, in both unadjusted (*B* = − 0.05, SE = 0.02, OR 0.95, 95% CI 0.92–0.98, *p* = 0.002; Table 2) and adjusted models (*B* = − 0.04, SE = 0.02, OR 0.96, 95% CI 0.93–0.996, *p* = 0.031). Moreover, we found a significant interaction between BMI and age (*B* = − 0.003, SE = 0.001, OR 0.997, 95% CI 0.995–0.9996, *p* = 0.025), suggesting that the association between BMI and mortality was stronger in older patients than that in younger patients. However, gender and cause of ICU admission did not moderate the association between BMI and mortality (*p*s > 0.05; Additional file 1: Table S2).

**Table 2:** Relationship between BMI (continuous) and mortality

	Unadjusted model		Adjusted model		Adjusted model with interaction	
	OR (95% CIs)	*p* value	OR (95% CIs)	*p* value	R (95% CIs)	*p* value
Age	–	–	1.02 [1.01, 1.03]	< 0.001***	1.09 [1.03, 1.15]	0.004**
Sex (female)	–	–	0.75 [0.55, 1.03]	0.074	0.76 [0.56, 1.05]	0.091
Cholesterol	–	–	0.97 [0.87, 1.08]	0.529	0.96 [0.86, 1.06]	0.426
APACHE II score	–	–	1.10 [1.08, 1.12]	< 0.001***	1.10 [1.08, 1.13]	< 0.001***
Diabetes mellitus	–	–	0.76 [0.53, 1.08]	0.119	0.77 [0.54, 1.10]	0.148
Mean glucose level	–	–	1.07 [0.98, 1.16]	0.144	1.06 [0.98, 1.16]	0.153
Use of corticosteroids	–	–	3.62 [2.65, 4.94]	< 0.001***	3.64 [2.66, 4.98]	< 0.001***
BMI	0.95 [0.92, 0.98]	.002**	0.96 [0.93, 0.996]	0.031*	1.15 [0.98, 1.36]	0.088
BMI × age interaction	–	–	–	–	0.997 [0.995, 0.9996]	0.025*

*BMI* body mass index, *APACHE* *II* Acute Physiology and Chronic Health Evaluation II

*p < 0.05, **p < 0.01, ***p < 0.001

Next, to supplement the previous analyses, we conducted binary logistic regression with BMI included as a categorical variable (i.e., underweight, normal weight, overweight, and obese groups). Mortality rates were 43.15% in underweight, 30.00% in normal weight, 21.99% in overweight, and 26.32% in obese groups. We used the overweight group, which had the lowest mortality rate, as the comparison group. In unadjusted models, underweight (*B* = 0.99, SE = 0.22, OR 2.69, 95% CI 1.75–4.15, *p* < 0.001; Table 3) and normal weight patients (*B* = 0.42, SE = 0.17, OR 1.52, 95% CI 1.08–2.13, *p* = 0.015) had increased odds of death. Obese and overweight patients had similar odds of death (*B* = 0.24, SE = 0.26, OR 1.27, 95% CI 0.77–2.10, *p* = 0.356).

**Table 3:** Relationship between BMI groups and mortality

	Unadjusted model	Adjusted model	Adjusted model with interaction			
	OR (95% CIs)	*p* value	OR (95% CIs)	*p* value	OR (95% CIs)	*p* value
Age	–	–	1.02 [1.01, 1.03]	< 0.001***	1.00 [0.98, 1.02]	0.982
Sex (female)	–	–	0.74 [0.54, 1.01]	0.058	0.74 [0.54, 1.02]	0.064
Cholesterol	–	–	0.97 [0.87, 1.08]	0.598	0.96 [0.87, 1.07]	0.477
APACHE II score	–	–	1.10 [1.08, 1.13]	< 0.001***	1.10 [1.08, 1.13]	< 0.001***
Diabetes mellitus	–	–	0.76 [0.53, 1.08]	0.124	0.77 [0.54, 1.10]	0.144
Mean glucose level	–	–	1.08 [0.99, 1.17]	0.090	1.07 [0.99, 1.17]	0.101
Use of corticosteroids	–	–	3.61 [2.64, 4.93]	< 0.001***	3.65 [2.66, 5.01]	< 0.001***
BMI						
Underweight	2.69 [1.75, 4.15]	< 0.001***	2.38 [1.43, 3.95]	< 0.001***	0.34 [0.03, 3.45]	0.364
Normal weight	1.52 [1.08, 2.13]	0.015*	1.29 [0.88, 1.89]	0.185	0.21 [0.03, 1.28]	0.091
Obese	1.27 [0.77, 2.10]	0.356	1.08 [0.61, 1.90]	0.790	0.85 [0.07, 11.11]	0.903
BMI × age interaction						
Underweight	–	–	–	–	1.03 [0.995, 1.07]	0.089
Normal weight	–	–	–	–	1.03 [1.001, 1.06]	0.045*
Obese	–	–	–	–	1.00 [0.96, 1.05]	0.868

*BMI* body mass index; *APACHE* *II* Acute Physiology and Chronic Health Evaluation II

*p < 0.05, **p < 0.01, ***p < 0.001

**Fig. 1:**
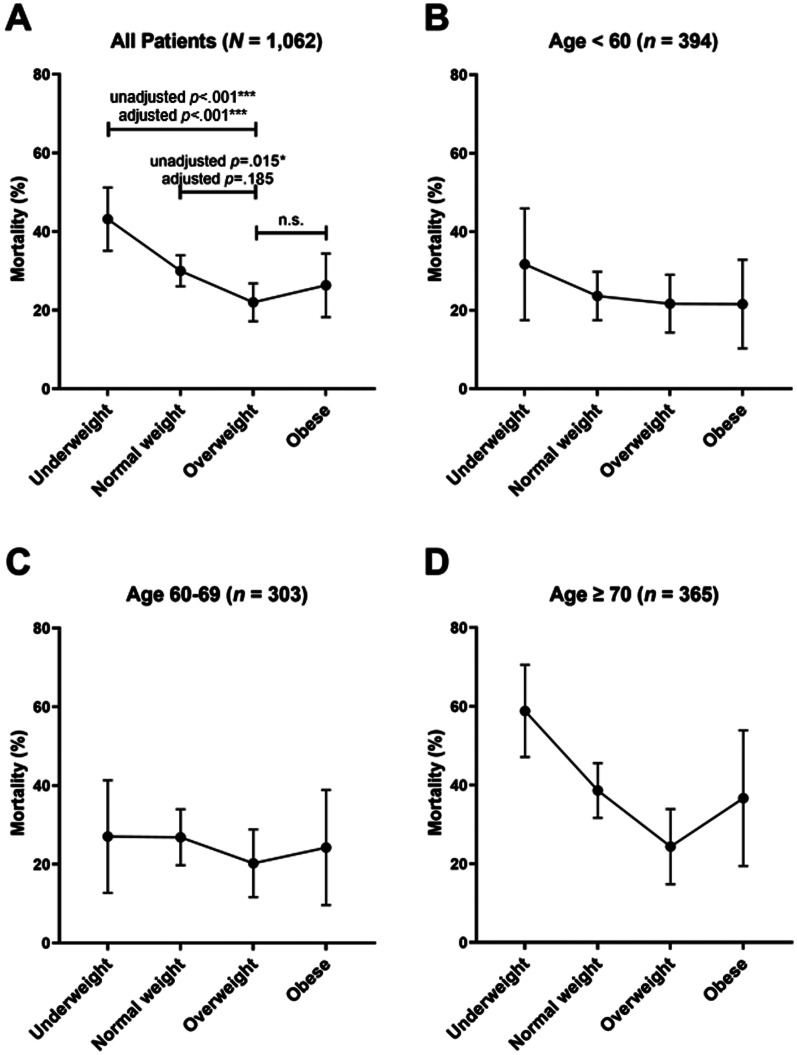
Mortality rates in each BMI group among all patients (**a**), patients below 60 years (**b**), patients between 60 and 69 years (**c**), and patients aged 70 or above (**d**). Error bars represent 95% confidence intervals. The depicted mortality rates are unadjusted mortality rates. Underweight is defined as BMI below 18.5 kg/m^2^, normal weight is defined as BMI between 18.5 and 23.99 kg/m^2^, overweight is defined as BMI between 24 and 27.99 kg/m^2^, and obese is defined as BMI ≥ 28 kg/m^2^. *p < 0.05, **p < 0.01, ***p < 0.001

After adjusting for covariates, underweight patients (*B* = 0.87, SE = 0.26, OR 2.38, 95% CI 1.43–3.95, *p* < 0.001) had greater odds of death, but normal weight (*B* = 0.26, SE = 0.19, OR 1.29, 95% CI 0.88–1.89, *p* = 0.185) and obese patients (*B* = 0.08, SE = 0.29, OR 1.08, 95% CI 0.61–1.90, *p* = 0.790) had similar odds of death, compared to overweight patients (Fig. 1a). The difference in mortality between underweight and overweight patients (*B* = 0.03, SE = 0.02, OR 1.03, 95% CI 0.995–1.07, *p* = 0.089) and the difference between obese and overweight patients (*B* = 0.003, SE = 0.02, OR 1.00, 95% CI 0.96–1.05, *p* = 0.868) did not interact with age. However, the advantage of being overweight (compared to normal weight) was moderated by age, such that being overweight is more advantageous in older adults (*B* = 0.03, SE = 0.01, OR 1.03, 95% CI 1.001–1.06, *p* = 0.045). In Fig. 1b–d, we categorized patients into three age groups (< 60, 60–69, ≥ 70) to depict the interaction effect. In addition, we found that gender and cause of ICU admission did not moderate the association between BMI groups and mortality (Additional file 1: Table S2).

### Higher glucose variability is associated with higher mortality

Higher GV was related to female sex [*t*(1060) = 3.23, *p* = 0.001], diabetes mellitus [*t*(1060) = 7.08, *p* < 0.001], and higher mean glucose level [*r*(1060) = 0.40, *p* < 0.001]. GV was not related to age [*r*(1060) = 0.04, *p* = 0.165], APACHE II score [*r*(1059) = 0.06, *p* = 0.052], total cholesterol [*r*(1060) = 0.04, *p* = 0.154], or the use of corticosteroids [*t*(1060) = 1.87, *p* = 0.062].

**Fig. 2:**
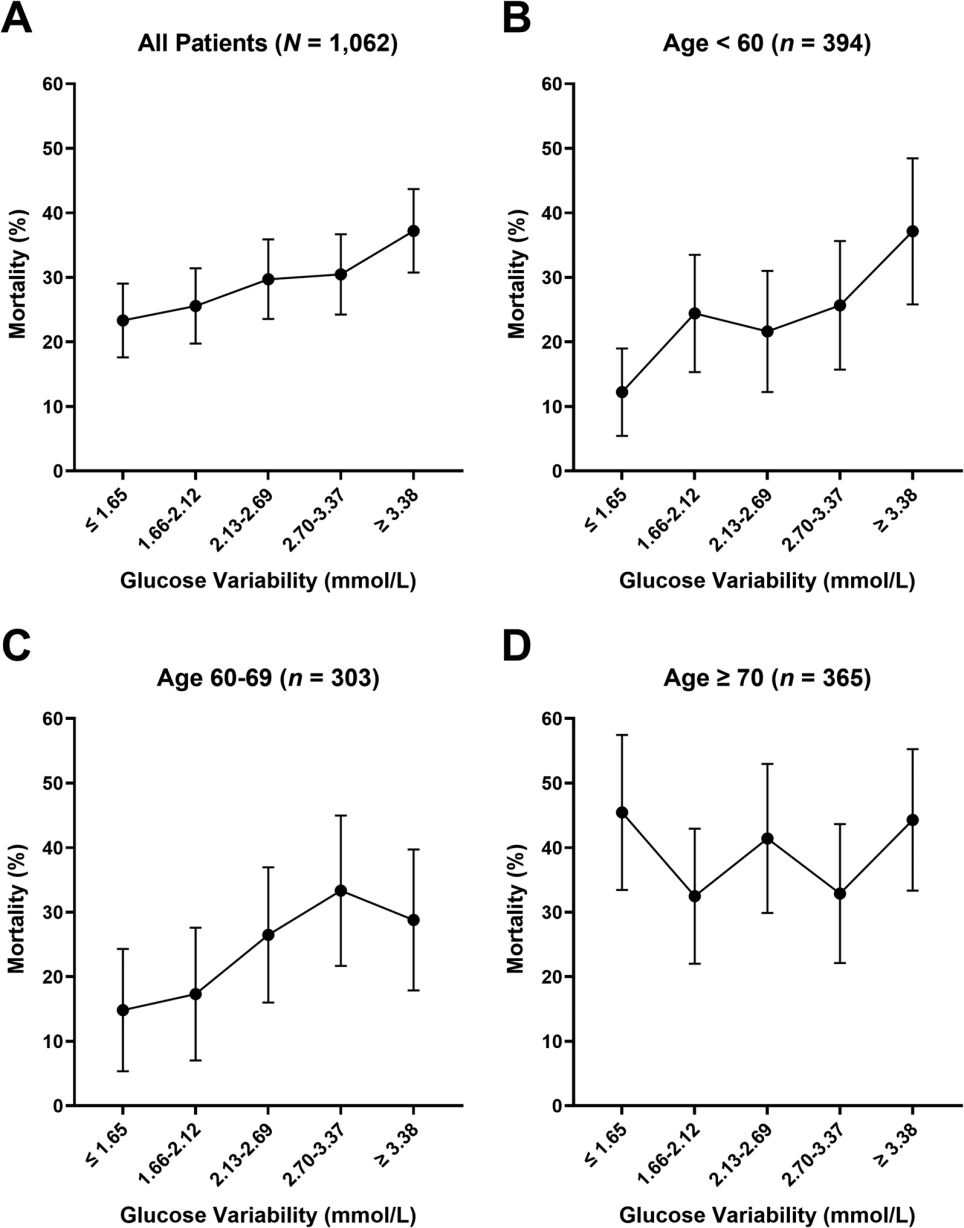
Mortality rates in each glucose variability quintile groups among all patients (**a**), patients below 60 years (**b**), patients between 60 and 69 years (**c**), and patients aged 70 or above (**d**). Error bars represent 95% confidence intervals. The depicted mortality rates are unadjusted mortality rates

Besides lower BMI, higher GV was associated with higher mortality in both the unadjusted (*B* = 0.14, SE = 0.06, OR 1.15, 95% CI 1.03–1.29, *p* = 0.011; Table 4) and adjusted models (*B* = 0.20, SE = 0.07, OR 1.23, 95% CI 1.06–1.42, *p* = 0.005). Figure 2a illustrates the mortality rate for each quintile group of GV.

**Table 4:** Relationship between glucose variability and mortality

	Unadjusted model		Adjusted model		Adjusted model with interaction	
	OR (95% CIs)	*p* value	OR (95% CIs)	*p* value	OR (95% CIs)	*p* value
Age	–	–	1.02 [1.01, 1.03]	< 0.001***	1.05 [1.02, 1.08]	< 0.001***
Sex (female)	–	–	0.76 [0.55, 1.03]	0.079	0.75 [0.55, 1.03]	0.072
Cholesterol	–	–	0.95 [0.86, 1.06]	0.359	0.95 [0.86, 1.06]	0.357
APACHE II score	–	–	1.10 [1.08, 1.12]	< 0.001***	1.10 [1.08, 1.12]	< 0.001***
Diabetes mellitus	–	–	0.70 [0.50, 1.001]	0.051	0.71 [0.50, 1.01]	0.058
Mean glucose level	–	–	1.00 [0.92, 1.10]	0.944	1.00 [0.92, 1.10]	0.925
Use of corticosteroids	–	–	3.66 [2.68, 5.00]	< 0.001***	3.67 [2.68, 5.01]	< 0.001***
Glucose variability	1.15 [1.03, 1.29]	0.011*	1.23 [1.06, 1.42]	0.005**	2.60 [1.33, 5.07]	0.005**
Glucose variability × age interaction	–	–	–	–	0.99 [0.98, 0.999]	0.025*

*BMI* body mass index; *APACHE* *II* Acute Physiology and Chronic Health Evaluation II

*p < 0.05, **p < 0.01, ***p < 0.001

Age was unrelated to mean glucose [*r*(1060) = 0.03, *p* = 0.319], GV [*r*(1060) = 0.04, *p* = 0.165], or the occurrence of hypoglycemia [*t*(1060) = 1.09, *p* = 0.275]. However, we found a significant interaction between age and GV on mortality (*B* = − 0.01, SE = 0.01, OR 0.99, 95% CI 0.98–0.999, *p* = 0.025), such that the association between GV and mortality attenuated with increasing age. In Fig. 2b–d, we depicted the interaction effect by categorizing patients into three age groups (< 60, 60–69, ≥ 70). To eliminate the potential confounding effect of hypoglycemia on this moderation effect, we additionally conducted sensitivity analysis with *n* = 920 individuals who did not experience hypoglycemia (at least one blood glucose measurement ≤ 3.9 mmol/L) during ICU stay. The interaction between age and GV on mortality stays significant, after controlling for covariates (*B* = –0.01, SE = 0.01, OR 0.99, 95% CI 0.98–0.999, *p* = 0.031). Last, the interactions between gender/cause of ICU admission and GV on mortality were non-significant (Additional file 1: Table S3).

## Discussion

We found that while higher BMI groups had lower mortality rates (underweight 43.15%, normal 30%, overweight 21.99%), this trend did not continue into the obese group (mortality 26.32%). Such results suggest a U-shape association between BMI groups and hospital mortality in ICU patients, consistent with previous research [2, 8, 10, 20]. Furthermore, this obesity paradox was stronger in older patients, comparing to their younger counterparts. In addition to high BMI, we found that lower GV was associated with lower mortality among all patients, and this association became weaker as age increased.

Underweight patients had the poorest prognosis, independent of covariates. This phenomenon might be explained by the cachexia caused by chronic comorbidities, malnutrition status, or cancer [21]. In addition, muscle wasting and loss of appetite caused by aging may also contribute to the high mortality in underweight patients [22–25]. According to the reserve fuel hypothesis, adipose tissue can provide energy reserve for a patient undergoing physiological stress due to either chronic or acute illness [26]. Therefore, underweight patients had the highest mortality rate because of their low energy reserve. However, the similar mortality rates between the normal weight and the obese groups cannot be fully explained by the reserve fuel hypothesis. One potential explanation is that while the obese patients benefited from having more adipose tissue, they also suffered more from chronic inflammation, comparing to normal weight patients [7]. Previous studies revealed that chronic inflammation exists commonly among obese patients and is associated with poor prognosis [1, 26].

It is noteworthy that the association between obesity and mortality is stronger in older patients in our study. Similar findings were reported in some recent studies [10, 11]. For example, Fukuoka et al. (2019) found a significant obesity paradox among older patients (age ≥ 70 years), but not among younger patients with acute myocardial infarction after percutaneous coronary intervention. There are two potential explanations for this finding. First, elderly patients with high BMI are expected to be more resilient to the deleterious effect of obesity than younger patients, due to survival bias [11]. Second, older patients may have higher composition of adipose tissue than younger patients with the same BMI [23], which strengthened the association between BMI and mortality.

Similar to the current study, multiple observational studies consistently reported an association between GV and hospital mortality in ICU patients [27–29]. GV causes damage to patients through unique mechanisms, which is independent from hyperglycemia or hypoglycemia. For example, both in vitro and in vivo experiments revealed that glucose fluctuations led to endothelial dysfunction via overproduction of oxidative stress metabolites [30]. In addition to the association between greater GV and higher mortality rate, the current study found that age plays a moderating role, where the association between GV and mortality becomes weaker as age increases. We postulate several potential mechanisms to explain this phenomenon.

One potential mechanism is that the oxidative damage caused by aging limits the deleterious effect of GV. According to the oxidative stress theory of aging, reactive oxygen species (ROS) accumulates progressively in the body as antioxidant protection diminished by time [31]. The mechanism of endothelial damage caused by GV also acts through ROS. According to the law of chemical equilibrium, the more ROS accumulated because of aging, the smaller amount of ROS would be formed as a result of GV. Therefore, it is possible that the fluctuations of glucose in older patients cannot result in the same level of damage as they do in younger patients.

Another potential mechanism is that the high prevalence of cardiovascular disease (CVD) and DM in older patients might conceal the effect of GV on mortality. Beta islet cells in the pancreas and other glucose regulating counterparts lose function progressively during aging [32]; thus older patients are more likely to develop DM. Epidemiology studies also reported a higher prevalence of DM in older patients [12, 33]. Furthermore, atherosclerotic microvascular damage progresses with time and CVD is the leading cause of death in the older population in China [34]. A recent study found that GV could not predict ICU mortality in diabetic patients, while there is a significant association between GV and ICU mortality in non-diabetic patients [35]. Since current evidence suggested that GV causes damage via vascular endothelial dysfunction [30, 36], there are some overlaps in the pathogenicity among GV, CVD, and DM. Thus, it is possible that the death caused by CVD and DM masked the effect of GV in older patients.

The current study has several strengths. First, our study used a large sample and this sample size ensured we have enough statistical power to detect small effects. Second, the age range of our sample is wide, from 17 to 96 years, which allowed us to investigate age as a moderator. Finally, we used complete glucose measurements throughout ICU stay to calculate GV, which allowed us to capture all the fluctuations in glucose levels throughout the different phases of ICU stay.

Limitations of the current study include the observational and retrospective nature, which does not allow us to assess causal relationships. Due to the complexity of patient care at ICU, the possibility of reverse causality exists in the relationship between GV and mortality cannot be ignored. However, we were not able to rule out this reverse causality due to our study design. Furthermore, our study sample was composed by Chinese residents (predominantly Asian). Hence, the findings may not be generalizable to other racial/ethnic groups. In addition, body composition and waist circumference were not measured, which limited our ability to investigate the relationship between different types of obesity and ICU mortality. Finally, oxidative stress markers were highly related to glucose metabolism and GV endothelial damage [30, 37], but they were not measured in our study. Therefore, we were not able to assess the association between oxidative stress and GV, or whether oxidative stress mediates the association between GV and mortality.

Future studies need to focus on the effect of body fat composition on mortality rate, in addition to BMI. Furthermore, future studies should investigate the underlying mechanisms of the interaction between GV and age on mortality, as well as identify the optimal glucose management strategies for different age groups. We encourage clinicians to consider the effect of BMI and GV when estimating the mortality in ICU.

## Conclusions

In summary, we demonstrated that, in a large cohort of critically ill patients, higher BMI is associated with lower hospital mortality and this association is stronger in older patients. Lower glucose variability is also associated with lower hospital mortality; however, with increasing age, the association between glucose variability and mortality becomes weaker. Future studies should investigate the underlying mechanisms of such phenomenon as well as the causal relationships between obesity, GV, and ICU mortality.

## Supplementary Information

**Additional file 1: Table S1.** ICU sliding scale insulin protocol. **Table S2.** The Interaction between BMI/glucose variability and Sex. **Table S3.** The Interaction between BMI/glucose variability and Cause of ICU Admission.

## Data Availability

The data sets generated and/or analyzed during the current study are available in the Open Science Framework repository (https://osf.io/4h2dq/).

## Abbreviations

APACHE II: Acute Physiology and Chronic Health Evaluation II score
BMI: Body mass index
CI: Confidence interval
CRRT: Continuous renal replacement therapy
CVD: Cardiovascular disease
DM: Diabetes mellitus
ICU: Intensive Care Unit
GV: Glucose variability
LDL: Low-density lipoproteins
HDL: High-density lipoproteins
OR: Odds ratio
ROS: Reactive oxygen species
SD: Standard deviation
SE: Standard error
