# Supplementary material for: Age moderates the relationships between obesity, glucose variability, and intensive care unit mortality: a retrospective cohort study
Source: J Intensive Care. 2021 Oct 26;9:68. doi: 10.1186/s40560-021-00582-4 (PMC8549309; doi:10.1186/s40560-021-00582-4)
Supplement: Supplementary file 1 — Additional file 1: Table S1. ICU sliding scale insulin protocol. Table S2. The Interaction between BMI/glucose variability and Sex. Table S3. The Interaction between BMI/glucose variability and Cause of ICU Admission. [file 40560_2021_582_MOESM1_ESM.docx]

**Age moderates the relationships between obesity, glucose variability, and Intensive Care Unit mortality: A retrospective cohort study**

Lusi Lu,^†1^ Yifeng Lu,^†2^ Chenlu Gao,^3,4^ & Nan Zhang*^1^

^1^Department of Endocrinology, Sir Run Run Shaw Hospital, School of Medicine, Zhejiang University (3 Qingchun E Rd, Hangzhou, Zhejiang, China)

^2^Touro College of Osteopathic Medicine (60 Prospect Ave, Middletown, New York, 10940, USA)

^3^Division of Sleep and Circadian Disorders, Brigham and Women’s Hospital 75 Francis St, Boston, Massachusetts, 02115, USA)

^4^Division of Sleep Medicine, Harvard Medical School (221 Longwood Ave, Boston, Massachusetts, 02115, USA)

^†^Contributed equally and share first authorship

*Correspondence: Nan Zhang (3198047@zju.edu.cn), Department of Endocrinology, Sir Run Run Shaw Hospital, School of Medicine, Zhejiang University (3 Qingchun E Rd, Hangzhou, Zhejiang, China)

Table S1. ICU sliding scale insulin protocol

| Blood Glucose Level (mmol/L) | Insulin Dose (iu) | Route |
| --- | --- | --- |
| 10.1 − 11.1 | 4 | Subcutaneous |
| 11.2 – 13.9 | 6 | Subcutaneous |
| 14.0 – 16.7 | 8 | Subcutaneous |
| 16.8 – 19.4 | 10 | Subcutaneous |
| <3.9 or > 19.4 | Based on physician’s judgment |  |

Table S2. The Interaction between BMI/glucose variability and Sex

|  | Model A | | Model B | | Model C | |
| --- | --- | --- | --- | --- | --- | --- |
|  | OR (95% CIs) | p-value | OR (95% CIs) | p-value | OR (95% CIs) | p-value |
| Age | 1.02 [1.01, 1.03] | <.001*** | 1.02 [1.01, 1.03] | <.001*** | 1.02 [1.01, 1.03] | <.001*** |
| Sex (female) | 0.93 [0.17, 5.22] | .938 | 0.96 [0.50, 1.83] | .896 | 0.81 [0.37, 1.73] | .580 |
| Cholesterol | 0.97 [0.87, 1.08] | .535 | 0.97 [0.87, 1.08] | .603 | 0.95 [0.86, 1.06] | .354 |
| APACHE II score | 1.10 [1.08, 1.12] | <.001*** | 1.10 [1.08, 1.13] | <.001*** | 1.10 [1.08, 1.12] | <.001*** |
| Diabetes mellitus | 0.76 [0.53, 1.07] | .118 | 0.76 [0.54, 1.09] | .135 | 0.70 [0.50, 1.001] | .051 |
| Mean glucose level | 1.07 [0.98, 1.16] | .144 | 1.08 [0.99, 1.17] | .086 | 1.00 [0.92, 1.10] | .950 |
| Use of corticosteroids | 3.61 [2.65, 4.93] | <.001*** | 3.63 [2.65, 4.97] | <.001*** | 3.66 [2.68, 5.00] | <.001*** |
| BMI (continuous) | 0.96 [0.92, 1.01] | .111 | -- | -- | -- | -- |
| BMI (continuous) × Sex interaction | 0.99 [0.92, 1.07] | .801 | -- | -- | -- | -- |
| BMI (categorical) | -- | -- | NA | .064 | -- | -- |
| BMI (categorical) × Sex interaction | -- | -- | NA | .736 | -- | -- |
| Glucose variability | -- | -- | -- | -- | 1.24 [1.03, 1.49] | .023* |
| Glucose variability × Sex interaction | -- | -- | -- | -- | 0.98 [0.75, 1.27] | .857 |

In Model A, we tested the interaction between BMI (as a continuous variable) and sex on mortality.

In Model B, we tested the interaction between BMI (as a categorical variable) and sex on mortality.

In Model C, we tested the interaction between glucose variability and sex on mortality.

Note. For categorical predictors with more than 2 levels, we present the p-values for the omnibus test.

**p*<.05, ***p*<.01, ****p*<.001.

BMI = body mass index; APACHE II = Acute Physiology and Chronic Health Evaluation II.

Table S3. The Interaction between BMI/glucose variability and Cause of ICU Admission

|  | Model A | | Model B | | Model C | |
| --- | --- | --- | --- | --- | --- | --- |
|  | OR (95% CIs) | p-value | OR (95% CIs) | p-value | OR (95% CIs) | p-value |
| Age | 1.02 [1.01, 1.03] | <.001*** | 1.02 [1.01, 1.03] | .001** | 1.02 [1.01, 1.03] | <.001*** |
| Sex (female) | 0.74 [0.54, 1.02] | .062 | 0.71 [0.51, 0.98] | .040* | 0.74 [0.54, 1.02] | .065 |
| Cholesterol | 0.96 [0.86, 1.07] | .448 | 0.96 [0.86, 1.08] | .481 | 0.94 [0.84, 1.05] | .260 |
| APACHE II score | 1.10 [1.08, 1.12] | <.001*** | 1.10 [1.08, 1.13] | <.001*** | 1.10 [1.08, 1.12] | <.001*** |
| Diabetes mellitus | 0.71 [0.49, 1.01] | .058 | 0.71 [0.50, 1.03] | .071 | 0.66 [0.46, 0.95] | .025* |
| Mean glucose level | 1.08 [0.99, 1.18] | .069 | 1.10 [1.003, 1.19] | .043* | 1.01 [0.92, 1.11] | .854 |
| Use of corticosteroids | 2.97 [2.12, 4.16] | <.001*** | 2.97 [2.11, 4.18] | <.001*** | 2.83 [2.02, 3.97] | <.001*** |
| Cause for ICU admission | NA | .635 | NA | .776 | NA | .027* |
| BMI (continuous) | 0.96 [0.87, 1.05] | .332 | -- | -- | -- | -- |
| BMI (continuous) × cause for ICU admission interaction | NA | .842 | -- | -- | -- | -- |
| BMI (categorical) | -- | -- | NA | .552 | -- | -- |
| BMI (categorical) × cause for ICU admission interaction | -- | -- | NA | .830 | -- | -- |
| Glucose variability | -- | -- | -- | -- | 1.42 [1.08, 1.88] | .013* |
| Glucose variability × cause for ICU admission interaction | -- | -- | -- | -- | NA | .319 |

In Model A, we tested the interaction between BMI (as a continuous variable) and cause of ICU admission on mortality.

In Model B, we tested the interaction between BMI (as a categorical variable) and cause of ICU admission on mortality.

In Model C, we tested the interaction between glucose variability and cause of ICU admission on mortality.

Note. For categorical predictors with more than 2 levels, we present the p-values for the omnibus test.

**p*<.05, ***p*<.01, ****p*<.001.

BMI = body mass index; APACHE II = Acute Physiology and Chronic Health Evaluation II.
